# Unveiling Exciton‐Plasmon Polariton Coupling Regions via Polarization‐Enhanced Optical Nanoscopy

**DOI:** 10.1002/advs.202507822

**Published:** 2025-08-11

**Authors:** Bin Chan Joo, Dong Hee Park, Kyu Ri Choi, Yeon Ui Lee

**Affiliations:** ^1^ Department of Physics Chungbuk National University Cheongju Chungbuk 28644 Republic of Korea

**Keywords:** exciton‐plasmon polariton coupling, light‐matter interaction, polarization, super‐resolution optical imaging

## Abstract

Nanoscale accuracy in single‐molecule localization is a crucial function in wide‐field super‐resolution optical microscopy by surpassing the diffraction limit. However, achieving high localization accuracy remains a challenge due to limitations in the signal‐to‐noise ratio and the complexity of molecular environments. In this study, a novel polarization‐enhanced single‐molecule localization microscopy (P‐SMLM) technique is introduced, incorporating dynamic polarization modulation to enhance the localization accuracy significantly. By modulating the polarization state of the excitation light, the technique leverages molecular sparsity, enabling more precise position determination. A 16 fold improvement in localization accuracy is shown experimentally compared to conventional methods, particularly under low signal‐to‐noise conditions. Moreover, the P‐SMLM enables direct visualization of exciton‐plasmon polariton coupling regions at room temperature. This findings highlight the potential of polarization modulation as a versatile tool for advancing single‐molecule localization microscopy (SMLM) accuracy and its applicability in diverse scientific and technological fields.

## Graphical abstract

Polarization‐enhanced single‐molecule localization microscopy, utilizing a liquid crystal variable retarder (LCVR), actively controls the on‐off intensity modulation of fluorophores. This active intensity modulation improves the localization accuracy of the point spread function (PSF), enabling high‐contrast super‐resolution imaging with significantly fewer image frames. Polarization‐modulated super‐resolution imaging reveals the spatial scale at which exciton–plasmon polariton coupling occurs.

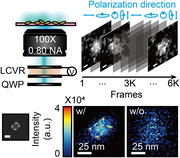

## 1 Introduction

Recent advancements in optical imaging techniques, including multicolor fluorescence microscopy^[^ ^1^ ^]^, time‐resolved fluorescence microscopy^[^ ^2^, ^3^ ^]^, and dark‐field microscopy^[^ ^4^ ^]^, have attempted to visualize light‐matter interactions with greater detail. Nonetheless, these methods remain constrained by Abbe's diffraction limit, hindering their ability to define sub‐wavelength light‐matter interaction zones with precision. Understanding and visualizing light‐matter interactions at the nanoscale is central to fields such as nanophotonics, materials science, and bioimaging. These interactions govern key processes, including fluorescence emission, energy transfer, and plasmonic enhancement, and their spatial characterization is essential for both fundamental studies and device applications. Single‐molecule localization microscopy (SMLM)^[^ ^5^, ^6^, ^7^ ^]^ has revolutionized fluorescence imaging by overcoming the diffraction limit, allowing nanoscale resolution in biological and material sciences. SMLM‐based techniques, such as stochastic optical reconstruction microscopy (STORM),^[^ ^8^ ^]^ points accumulation for imaging in nanoscale topography (PAINT),^[^ ^9^ ^]^ and Brownian optical microscopy,^[^ ^10^, ^11^ ^]^ rely on localizing the centroid of the point‐spread function (PSF) of individual fluorescent molecules. With its ability to achieve resolution beyond 20 nm, SMLM has opened new avenues in studying nanoscale biological processes and material interfaces.

Despite their success, these techniques face inherent limitations: the statistical randomness in localizing single molecules requires a large number of frames to reconstruct super‐resolved images.^[^ ^7^, ^12^, ^13^ ^]^ Common approaches include chemical modifications or the introduction of external agents, which can chemically induce the transition of fluorophores between fluorescent and non‐fluorescent states.^[^ ^14^, ^15^ ^]^ This not only prolongs image acquisition but also increases the susceptibility of fluorescent dyes to photodamage during extended laser exposure.^[^ ^16^, ^17^ ^]^ Moreover, the dependence on specific fluorescent probes with suitable photostability and emission properties imposes additional restrictions, further emphasizing the need to reduce the number of frames while maintaining high localization accuracy.

One promising approach to address this challenge is the controlled modulation of fluorescence intensity^[^ ^18^, ^19^ ^]^ in SMLM. By actively manipulating the blinking behavior of fluorophores—switching between their on and off states—it becomes possible to reduce the number of frames required, adding sparsity to each recording while enhancing localization precision.^[^ ^8^, ^20^, ^21^ ^]^ Improved emission intensity and blinking control ensure better distinguishability of individual molecules, enabling more precise visualization of nanoscale regimes where the kinetics of fluorophores are actively controlled. Recently, leveraging light‐matter interaction has emerged as a promising alternative for actively controlling fluorescence dynamics without causing chemical perturbations or irreversible damage.

Light‐matter interactions play a fundamental role in various applications, including semiconductor quantum dots, 2D materials,^[^ ^22^ ^]^ lasers,^[^ ^23^ ^]^ and optical communications.^[^ ^24^ ^]^ Among these, exciton‐plasmon polariton coupling systems offer unique opportunities to control and enhance light emission properties through the Purcell effect,^[^ ^25^, ^26^, ^27^ ^]^ which confines electromagnetic modes within plasmonic nanostructures. This effect enhances emission rates in a polarization‐dependent manner,^[^ ^10^, ^28^, ^29^ ^]^ enables spectral manipulation, and provides insights into the dynamics of molecular excitons in complex environments.

In this study, we introduce polarization‐enhanced single‐molecule localization microscopy (P‐SMLM), a super‐resolution imaging technique that employs polarization modulation induced by exciton‐plasmon polariton coupling within a light‐matter interaction system. By integrating a liquid crystal variable retarder (LCVR) into a standard confocal microscopy setup, P‐SMLM dynamically manipulates fluorophore emission through precisely controlled polarization states. This modulation enhances the probability of alignment between the excitation polarization and the fluorophore dipole orientation, resulting in pronounced, spatially resolved blinking signals and improved contrast between the fluorophore on and off states. Unlike conventional methods, this does not rely on complex photochemical switching mechanisms, enabling sparse fluorophore activation with fewer required imaging frames. As a result, P‐SMLM minimizes potential photodamage and enhances overall imaging efficiency.

This approach not only improves localization accuracy but also facilitates the mapping of interaction zones, such as exciton‐plasmon polariton coupling regions, that are otherwise inaccessible using conventional imaging techniques. Furthermore, because the underlying mechanism is based on the universal dipole characteristics of fluorophores and their polarization‐dependent excitation efficiency, P‐SMLM can be broadly applied to various nanostructured materials and biological systems where polarization‐resolved responses arise. Its adaptability across different sample types highlights its potential as a general‐purpose super‐resolution method for exploring nanoscale optical phenomena in both physical and life sciences.

Consequently, P‐SMLM significantly increases localization accuracy*—*by up to 16 fold*—*and enables precise visualization of complex nanoscale light‐matter interaction zones, and reduces the required number of imaging frames, thus minimizing potential photodamage and enhancing overall imaging efficiency.

Using P‐SMLM, we demonstrate super‐resolution imaging of the spontaneous emission enhancement around gold nanorods (AuNRs), which varied with the polarization direction of incident light. By enabling polarization‐controlled excitation of individual fluorescent molecules, P‐SMLM overcomes the limitations of fluorophore specificity associated with conventional methods. The resulting strong blinking signals and improved localization accuracy facilitated more precise visualization of nanoscale light‐matter interaction regions, such as exciton‐plasmon coupling zones, typically inaccessible by traditional imaging techniques.

## 2 Results

**Figure 1:**
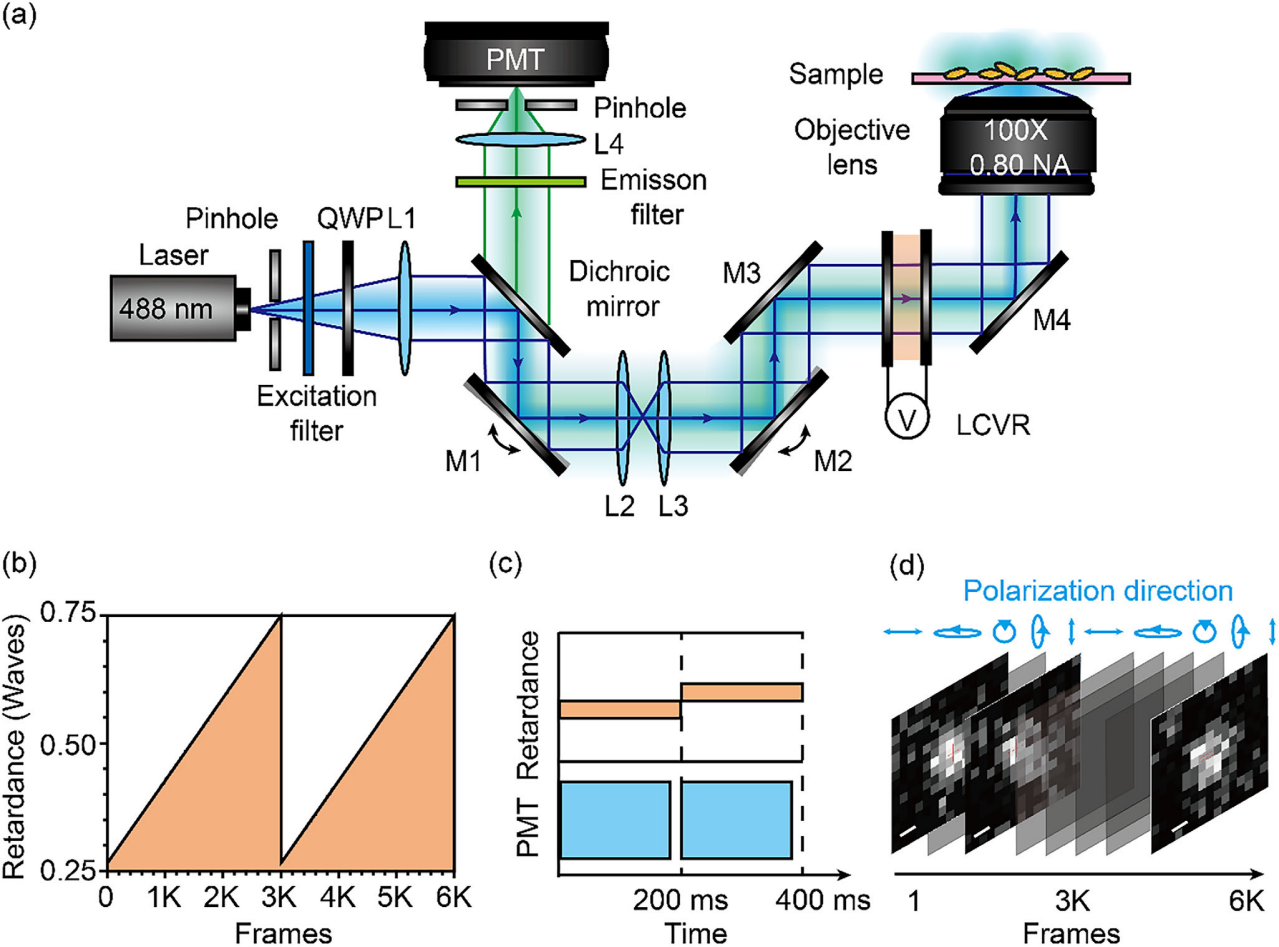
Schematic illustration of polarization‐enhanced single‐molecule localization microscopy (P‐SMLM). a) The P‐SMLM is based on a home‐modified confocal microscope. The polarization state of the 488 nm excitation laser beam is controlled using a quarter‐wave plate (QWP) and a liquid crystal variable retarder (LCVR). The excitation beam is focused onto the sample plane with varying polarization states. A series of fluorescence images obtained under actively controlled polarization states is collected by a photomultiplier tube (PMT). b) The phase retardance of the LCVR is programmed by applying an external voltage during the acquisition of 6K frames. By sweeping the polarization of excitation and emission, the spatiotemporal distributions of emission are modulated accordingly, resulting in sparsity enhancement. c) A schematic of synchronization between LCVR and PMT to avoid signal mixing during the acquisition time is shown. d) A series of raw images obtained during retardance scanning. Scale bar: 170 nm. L: lens, M: mirror.

In contrast to conventional fluorescence polarization microscopy, which cannot distinguish neighboring fluorescent emitters within the optical diffraction limit, polarization modulation‐induced sparsity in single‐molecule localization enables super‐resolution imaging. As illustrated in **Figure** **1**a and P‐SMLM utilizes polarization modulation to increase the spatiotemporal sparsity of emitters.

A 488 nm linearly polarized continuous‐wave laser beam is converted into a circularly polarized beam by passing through a quarter‐wave plate (QWP), and is subsequently modulated by an LCVR, which is positioned in front of a 100× dry objective lens with a numerical aperture (NA) of 0.8. The excitation beam uniformly illuminates the sample from the substrate side at an intensity of 2.9 W cm^−2^ (see Experimental Section for experimental setup details). Fluorescence signals are collected by a photomultiplier tube (PMT) via a dichroic mirror and two distinct emission filters (500–550 nm and 590–650 nm), used in separate acquisition sessions.

Synchronization between the LCVR modulation and PMT signal acquisition ensures an accurate temporal correlation between the applied polarization state and the corresponding fluorescence emission. Programmable phase retardance is introduced to the excitation and emission beams through the external voltage control of the LCVR. Each image frame is acquired with a 180 ms exposure time, and a full polarization modulation cycle (0.5λ) comprises 6000 frames, with an LCVR dwell time of 200 ms per frame (Figure 1b,c).

By incrementally varying the LCVR retardance in steps of 0.0005λ per image frame, P‐SMLM generates enhanced blinking behavior from excited fluorophores whose dipole orientations are aligned with the incident electric field, resulting in improved spatiotemporal sparsity and enabling precise localization of emission events within nanoscale light‐matter interaction regions (Figure 1d). Polarization states at the sample plane were verified using an analyzer placed after the objective. A full sweep of ≈0.5 wave retardance ensured broad polarization coverage to maximize excitation sparsity across randomly oriented fluorophores.

**Figure 2:**
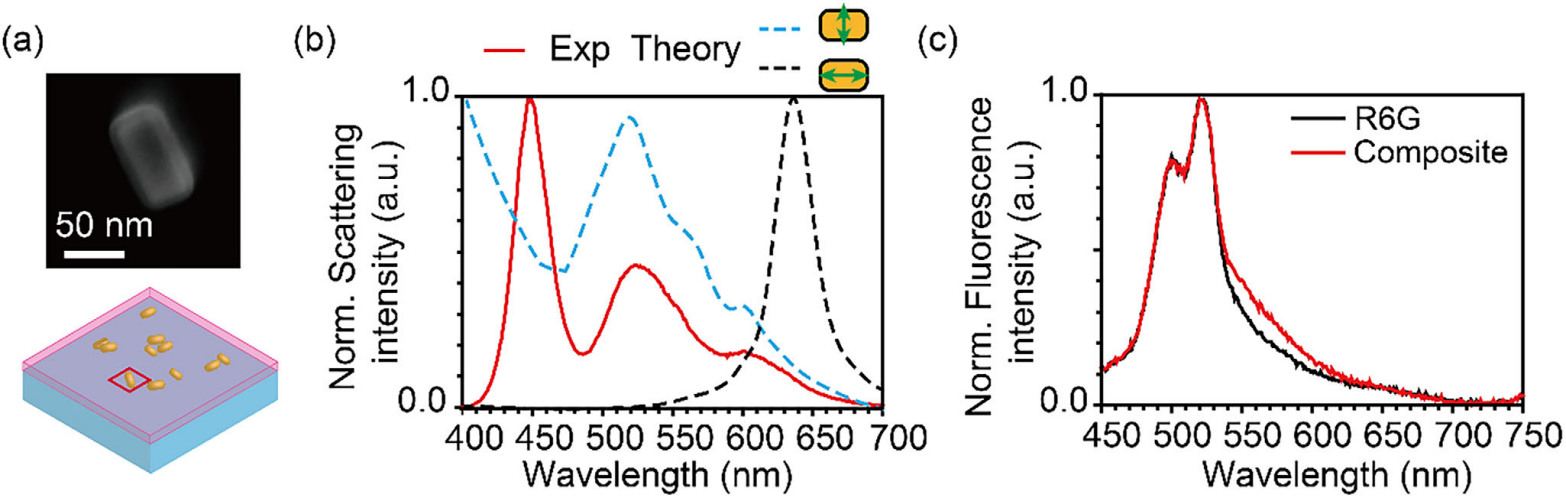
Optical characterization of the AuNR and the AuNR‐R6G composite system. a) SEM image of a single AuNR (top) and schematic of the AuNR–R6G composite film (bottom). The AuNRs are drop‐cast onto the R6G fluorescent thin film, where the R6G molecules are randomly oriented. Additional SEM images showing multiple AuNRs are provided in Figure S2 (Supporting Information). b) Normalized scattering spectra of randomly distributed AuNRs on a glass coverslip. The red solid line represents the scattering spectrum measured using a darkfield lens (100×, 0.90 NA), while the dashed lines represent the scattering spectra calculated through FDTD simulation. The blue and black dashed curves show the scattering spectra for incident polarization along the AuNR's longitudinal and transverse axes, respectively. c) Normalized fluorescence spectra of a pristine R6G film (black) and an AuNR‐R6G composite film (red).

Next, using P‐SMLM, we aim to achieve super‐resolution imaging of the spontaneous emission enhancement regime of fluorophores near the AuNRs, specifically in the exciton‐plasmon polariton coupled regime. The modified spontaneous emission behavior of fluorophores is influenced not only by the excitation polarization state but also by their interaction with the surrounding local electromagnetic environment.^[^ ^28^ ^]^ To control and enhance such emission, optical nanoresonators—such as photonic crystal microcavities and AuNRs—that confine light within subwavelength volumes are commonly employed. **Figure** **2**a shows an exciton–plasmon polariton coupling system, where gold nanorods (AuNRs; 25 nm diameter, 75 nm length, 25 nm thickness) are randomly embedded in a ≈40 nm‐thick rhodamine 6G (R6G) fluorescent molecular layer deposited on a glass coverslip. Detailed information regarding the sample preparation process is provided in the Experimental Section.

Figure 2b shows the measured scattering spectra (red solid curve) of randomly distributed AuNRs, revealing two broad resonance features. To identify the characteristic modes, finite‐difference time‐domain (FDTD) simulations were performed for a single AuNR, yielding distinct longitudinal (black dashed curve, ≈650 nm) and transverse (blue dashed curve, ≈530 nm) localized surface plasmon polariton (LSPP) resonance modes. The measured spectrum does not show two sharply resolved peaks as in the simulation, which can be attributed to the random orientation and distribution of AuNRs in the sample. The longitudinal mode appears weaker than expected due to the random orientation of AuNRs with respect to the incident polarization, which limits the effective excitation of their long axes. In such disordered configurations, the incident light interacts with nanorods at varying angles, leading to a mixed contribution of longitudinal and transverse modes, as well as higher‐order plasmonic resonances, which together result in the broad, overlapping spectral profile observed experimentally.

An absorption spectrum and an emission spectrum of the R6G thin film (Figure S1, Supporting Information) show substantial overlap with the LSPP resonance of the AuNRs. This overlap facilitates energy transfer between the LSPP and R6G excitons, resulting in the formation of an exciton‐plasmon polariton coupled system. In the weak coupling regime, the spontaneous emission properties of R6G excitons are modified by the Purcell effect, resulting in enhanced emission observed in the wavelength range of 500–650 nm, as shown in Figure 2c.

To identify the spatial region where the LSPPs generated by an AuNR can couple with the excitons of R6G, and to gain further insight into the spatial localization of fluorescence enhancement and the role of polarization, 3D FDTD simulations were conducted to mimic the experimental configurations and calculate the corresponding near‐field intensity distributions. The distributions shown in Figure S3 (Supporting Information) are separately obtained by averaging the electric field intensities over *xy*‐plane power monitors along the z‐axis at 4 nm intervals for excitation wavelengths of 525 and 620 nm, respectively. Note that the polarization orientation of the incident beam determines the excitation efficiency of R6G in the exciton‐plasmon polariton coupling regime. The simulations were subsequently compared with experimental results to evaluate the correspondence between predicted enhancement patterns and observed nanoscale emission distributions.

**Figure 3:**
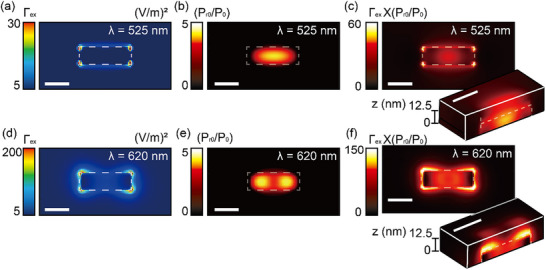
FDTD‐calculated excitation field intensity enhancement, radiative decay rate enhancement, and emission intensity enhancement. a–c) Excitation field intensity enhancement, Γ_*ex*_ = |*E*|^2^ /|*E*_0_|^2^, radiative decay rate enhancement, *P_r_*/*P*_*r*0_, and emission intensity enhancement, Γ_*ex*_(*P_r_*/*P*_*r*0_) at 525 nm and (d‐f) 620 nm, respectively, where E is the electric field intensity in the presence of a single AuNR, *E*_0_ is the electric field intensity in the absence of the nanorod, *P_r_*, *P*_*r*0_ is the power radiated into the free space in the absence of the AuNR. The inset figures show 3D mappings of the emission enhancement factor. The scale bar is 40 nm.

Next, the calculated fluorescence intensity enhancement around the AuNR is presented in **Figure** **3**. This enhancement is obtained by multiplying the excitation field enhancement by the radiative decay rate enhancement factor. The excitation field enhancement (Figure 3a,d) is calculated using an approach analogous to the experimental procedure used in P‐SMLM imaging, in which multiple fluorescence images are captured under varying incident polarization states. Accordingly, the excitation field distributions are evaluated at wavelengths of 525 and 620 nm, respectively. To calculate the radiative decay rate enhancement (Figure 3b,e), the emitting power enhancement generated by a point dipole source near the AuNR is spatially calculated by sweeping 5151 different source locations along the *x*‐, *y*‐, and *z*‐axes with 4 nm spacing within the region −100 nm ≤ *x* ≤ 100 nm, −50 nm ≤ *y* ≤ 50 nm, and 0 ≤ z ≤ 40 nm. For randomly oriented emitters, the emitting powers generated by a single dipole oriented along the *x*‐, *y*‐, and *z*‐axes are averaged from three separate simulations (see Experimental Section for FDTD simulation details). Although R6G has a high intrinsic quantum yield, fluorescence enhancement can still be achieved via excitation field enhancement near plasmonic nanostructures, as previously demonstrated.^[^ ^30^ ^]^

**Figure 4:**
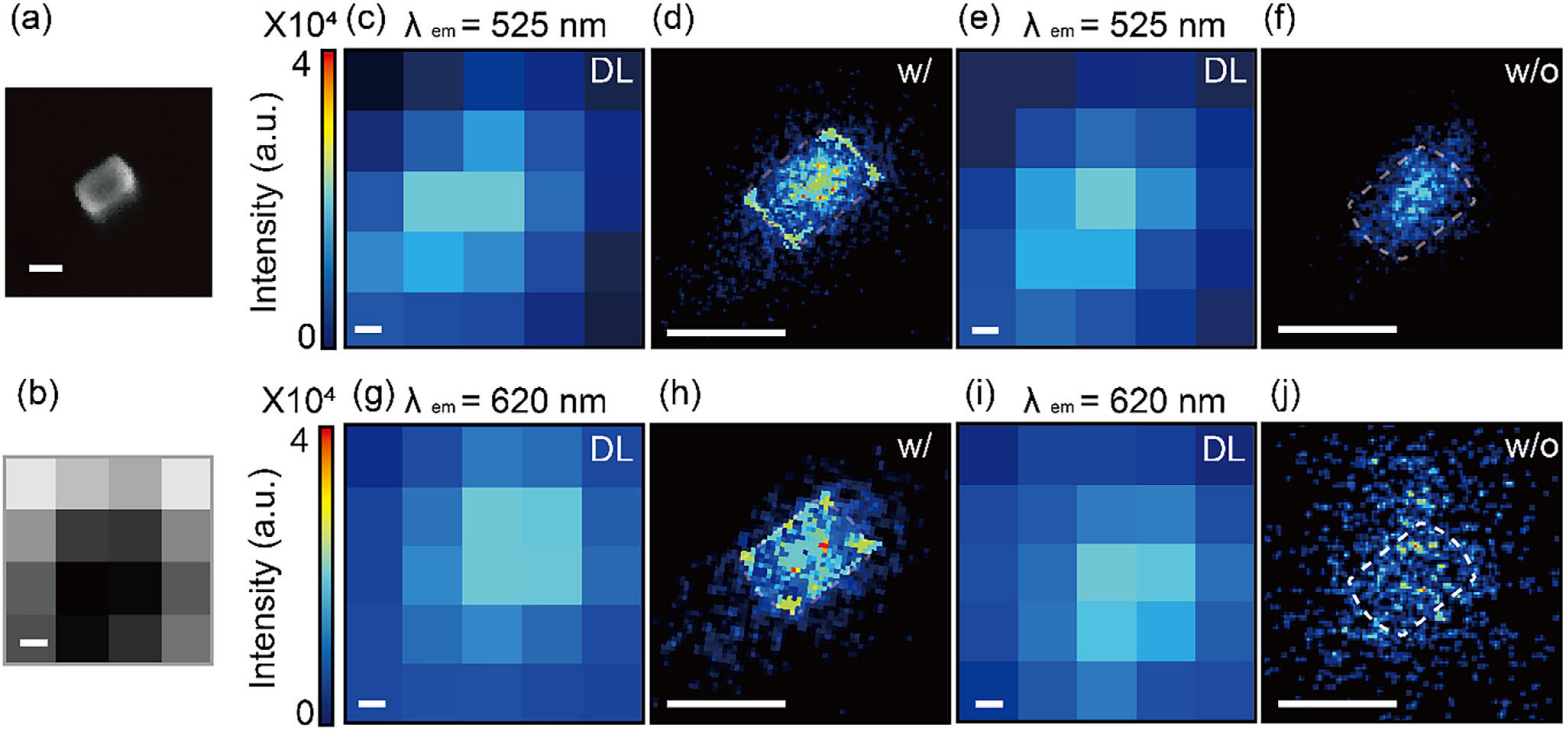
Visualization of exciton‐plasmon polariton interaction regimes. a) SEM image of the AuNR. b) Diffraction‐limited (DL) bright‐field optical microscopy image of the AuNR‐R6G composite structure. The dark center corresponds to the location of a single AuNR, which appears dark due to increased light scattering and absorption, resulting in reduced transmission under bright‐field illumination. c–f) DL and super‐resolution fluorescence images of exciton‐plasmon coupling zones at λ = 525 nm, with and without polarization modulation. g–j) DL and super‐resolution fluorescence images at λ = 620 nm, with and without polarization modulation. The dashed rectangles on the super‐resolution images represent the actual AuNR. The scale bar is 50 nm.

The fluorescence enhancement factors shown in Figure 3c,f at λ = 525 and 620 nm reveal the spatial regions where the fluorescence intensity of R6G is amplified, representing the exciton‐plasmon polariton coupling zones. The fluorescence images calculated from FDTD simulations (Figure 3c,f) and the experimental P‐SMLM images (**Figure** **4**) show good agreement. Building on these theoretical insights, experimental super‐resolution imaging using P‐SMLM was performed to resolve the spatially confined emission patterns near single AuNRs.

To experimentally achieve super‐resolution imaging of the exciton‐plasmon polariton coupled regime, we apply the following guiding principles for implementing a sparsity‐induced single‐molecule localization imaging technique using polarization modulation: 1) AuNRs support LSPPs excitation, which strongly confine the electric fields near the AuNRs under light excitation. 2) The molecular excitons of R6G interact with plasmon polaritons generated by AuNR, forming an exciton‐plasmon polariton coupled system.^[^ ^2^ ^]^ 3) The stochastic blinking of R6G molecules, induced by emission enhancement and quenching near the AuNRs^[^ ^19^, ^31^ ^]^ enables precise localization of fluorescent molecules. 4) Absorption and emission are maximized when the transition dipole moments of R6G molecules align parallel to the incident polarization.^[^ ^28^ ^]^ Therefore, as the polarization is modulated, the emission intensity varies spatially with nanoscale precision, generating abundant blinking signals. 5) A single fluorescence image is recorded for each specific single input polarization state. A total of 6000 diffraction‐limited raw images are acquired under 6000 modulated polarization states and subsequently processed using SMLM reconstruction in ImageJ^[^ ^31^ ^]^ to generate a single high‐contrast, super‐resolution fluorescence image.

A region containing a single AuNR was selected to visualize the exciton‐plasmon‐polariton coupling regime. The SEM image and bright‐field optical microscopy image are shown in Figure 4a,b, respectively. In the 500–550 nm emission range, the conventional fluorescence and P‐SMLM images of the single AuNR with polarization modulation are presented in Figure 4c,d, respectively, whereas the conventional fluorescence and SMLM images without polarization modulation are shown in Figure 4e,f. Similarly, for the 590–650 nm emission range, Figure 4g,h display the conventional fluorescence and P‐SMLM images with polarization modulation, while Figure 4i,j show the conventional fluorescence and SMLM images without polarization modulation. Notably, the P‐SMLM images (Figure 4d,h) reveal distinct, wavelength‐dependent interaction patterns: at 500–550 nm (Figure 4d), strong fluorescence signals appear at the short‐axis edges of the AuNR, whereas at 590–650 nm (Figure 4h), the signals are observed at the long‐axis edges. In contrast, the conventional SMLM images (Figure 4f,j) fail to reveal any significant spatial patterns. To assess the reproducibility of the observed localization improvement, we applied P‐SMLM to multiple AuNRs (Figure S6, Supporting Information). In all cases, polarization modulation led to consistent improvements in localization precision and resolution, confirming the robustness of the method.

**Figure 5:**
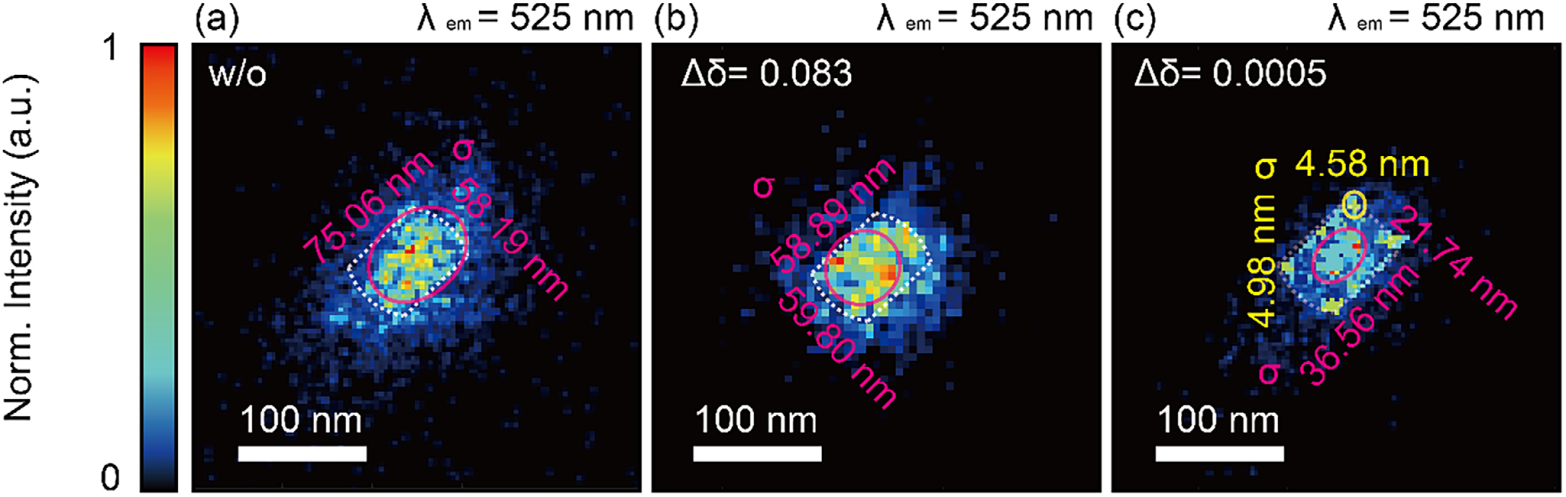
Polarization‐modulated SMLM images. Localization accuracy of the centroid positions of interaction hot spots under different polarization modulation conditions (retardance step Δδ): a) Δδ  =  0, b) Δδ  =  0.083λ, c) Δδ  =  0.0005λ. The standard deviation (*σ*) represents the relative hot spot size or the localization accuracy of the centroid position of a hot spot. Scale bar: 100 nm.

Indeed, the polarization‐modulated SMLM reconstruction improves image contrast by varying the incident light's polarization, thereby inducing strong blinking signals from individual R6G emitters (see Figure S4, Supporting Information) and enhancing localization accuracy. Figure S4 (Supporting Information) presents fluorescence intensity traces with and without polarization modulation, obtained by selecting a 10‐pixel‐wide region along the *x‐* and *y*‐axes centered on the AuNR and surrounding background. Fluorescence intensity is recorded frame‐by‐frame across 6000 images, enabling a detailed comparison between emission signals from the AuNR and background noise. **Figure** **5** shows the polarization modulation‐dependent localization accuracy of the centroid position of interaction hot spots. The standard deviation (σ) indicates the apparent size of each hot spot and quantifies localization precision. Polarization modulation enhances blinking behavior and improves the signal‐to‐noise ratio, thereby enabling super‐resolution imaging of individual nanoscale interaction zones. To further evaluate resolution enhancement, Fourier ring correlation (FRC) analysis is applied to P‐SMLM and conventional SMLM images. The resulting FRC‐based resolution is ≈26 nm for the P‐SMLM image and 41 nm for the conventional SMLM image, demonstrating a substantial improvement in spatial resolution achieved through polarization modulation (Figure S5, Supporting Information).

A 16‐fold improvement in localization accuracy is achieved with polarization modulation (Figure 5c), as indicated by a reduction in σ from 75.06 nm in the conventional case (Figure 5a) to 4.58 nm, particularly under low signal‐to‐noise ratio conditions. The reproducibility of these improvements is confirmed by applying P‐SMLM to multiple AuNRs (Figure S6, Supporting Information), consistently yielding enhanced localization precision and resolution across all cases, thereby validating the robustness of the technique. Collectively, these results demonstrate that polarization modulation not only improves localization precision but also enables direct mapping of interaction zones that are otherwise inaccessible using conventional SMLM techniques. These findings establish a foundation for broader applications in advanced fluorescence microscopy and plasmonic sensing, where precise control of light‐matter interactions is critical. Since P‐SMLM reduces the number of required frames while preserving localization accuracy, it can be applied broadly to fluorescence‐based super‐resolution imaging using various fluorescent probes, including those commonly used in live‐cell imaging.

## 3 Conclusion

We demonstrate polarization‐enhanced super‐resolution fluorescence imaging based on a single‐molecule localization microscopy approach. By employing an LCVR to modulate the polarization state of the excitation light, we achieved enhanced fluorescence contrast and improved localization accuracy through intense fluorescence fluctuations around the AuNR and effective background suppression during retardance scanning. To validate the experimental results, FDTD simulations were performed to generate ground truth images. The calculated fluorescence intensity enhancement, derived from the excitation field enhancement and radiative decay rate enhancement factors, closely matched the experimental P‐SMLM images, confirming the reliability of the polarization modulation strategy. The local field enhancement by AuNR and radiative emission enhancement of R6G emitters revealed the spatial distribution of the exciton‐plasmon polariton coupling region with nanoscale precision.

The P‐SMLM offers significant advantages for imaging phototoxic or photobleaching‐sensitive samples while enabling the visualization of nanoscale interaction zones beyond the diffraction limit. The technique revealed wavelength‐dependent interaction patterns, with fluorescence signals localized at specific regions of the AuNR, providing a powerful tool for investigating exciton‐plasmon coupling dynamics with high sensitivity and nanoscale resolution. These findings establish a foundation for broader applications in advanced fluorescence microscopy and plasmonic sensing, where precise control of light‐matter interactions is critical. Although this study highlights a specific use case involving plasmonic coupling, the polarization‐modulated SMLM approach can be extended to other systems, including orientation‐sensitive biomolecular imaging and non‐metallic nanostructures, where polarization‐dependent excitation governs fluorescence emission. This broader applicability positions the technique as a versatile strategy for nanoscale investigations in both materials science and biology. Future studies could extend this approach to other molecular emitters and more complex plasmonic architectures, further expanding its potential for high‐resolution imaging and sensing technologies. Beyond enhancing localization accuracy, polarization modulation may also enable selective imaging of emitters with distinct dipole orientations or polarization sensitivities, offering an additional contrast dimension for future multicolor or multidimensional super‐resolution applications.

## 4 Experimental Section

### Experimental Setup

A 488 ± 4 nm, 10 mW continuous‐wave diode laser (K1‐FLUO, Nanoscope System, Korea) served as the excitation source and was filtered through a 488/6 nm bandpass filter (FF01‐488/6‐25, Semrock), ensuring clean and narrowband excitation before entering the microscope. The linearly polarized beam was converted to circular polarization by a quarter‐wave plate (QWP), then modulated by a liquid crystal variable retarder (LCVR; LCC1513‐A, Thorlabs) placed in front of a 100× dry objective lens (NA 0.8, Olympus). The sample was illuminated via raster scanning from the substrate side with a scanned field of view of 87 µm × 87 µm, using an average irradiance of 2.9 W cm^−^^2^ (total laser power: 1.3 mW at the objective back aperture). Fluorescence emission was collected by a photomultiplier tube (PMT) through a dichroic mirror and either a 500–550 nm or 590–650 nm bandpass filter, depending on the acquisition session. The fluorescence images were obtained by point‐by‐point raster scanning, utilizing a resonant scanner for the fast axis and a galvanometer scanner for the slow axis. During scanning, fluorescence signals at each pixel were detected by a high‐quantum‐efficiency PMT equipped with a multialkali photocathode (Hamamatsu). Synchronization between LCVR modulation and PMT acquisition enabled accurate tracking of polarization‐resolved emission. Each 180 ms exposure frame corresponded to a specific retardance state, and one full polarization cycle (0.5λ) consisted of 6000 frames with 200 ms LCVR dwell time per frame.

### Sample Fabrication

The fluorescent film was prepared using Rhodamine 6G (Sigma Aldrich), dissolved in ethanol at a concentration of 5 mg mL^−1^. The solution was spin‐coated onto a 0.17 mm‐thick, pre‐cleaned coverslip using a PTFE filter (ADVANTEC, 11 mm diameter, 0.2 µm pore size). Spin‐coating was performed at 1000 rpm for 1 min to produce a 40 nm‐thick R6G film. A 20 *µ*L solution of AuNR dispersed in distilled water (Sigma–Aldrich) was mixed with 20 *µ*L of distilled water to ensure sparse distribution of the nanorods when drop‐cast on a substrate. The diluted AuNR solution was drop‐cast onto the pre‐spin‐coated R6G thin film. The size of AuNRs used in experiments is 25 nm in diameter, 75 nm in length, and 25 nm in thickness. To characterize the actual geometry, we measured over 50 individual nanorods from the same sample via SEM. The average length was (73.4 ± 4.8) nm, and the average diameter was (24.6 ± 2.1) nm, resulting in an average aspect ratio of ≈3.0 ± 0.4, consistent with the reported dimensions (25 × 75 nm). When a diluted AuNR solution is drop‐cast onto the R6G layer, partial dissolution occurs, allowing R6G molecules to adsorb randomly around the AuNRs.

### FDTD Simulations

All simulations were performed using the commercial software Ansys Lumerical FDTD Solutions (version 2022 R1.3), which does not require code sharing, as the simulation setup is conducted via GUI‐based input and standard solver modules. The simulated nanorod geometry was directly matched to the actual AuNR used in the experiments. Specifically, after fluorescence image acquisition, high‐resolution SEM images of the same region were obtained, and the nanorod responsible for the measured signal was identified. The shape, size, and orientation of this nanorod were then used as input for the FDTD simulation. The electric field intensity enhancement is independently calculated for linearly, elliptically, and circularly polarized plane waves incident on the AuNR from the glass substrate side and then averaged at wavelengths of 525 and 620 nm. For linearly polarized light, incident beams polarized along the *x*‐ and *y*‐axes are used. Elliptically polarized lights are generated by introducing phase retardance of −π/4 and π/4 between the *x*‐ and *y*‐polarized plane waves. Circularly polarized lights are generated by introducing phase retardance of −π/2 and π/2 between the *x*‐ and *y*‐polarized plane waves. The incident polarization was configured relative to the long axis of this specific AuNR, ensuring that the simulation results correspond closely to the actual experimental configuration. The boundary condition was set to a perfectly matched layer (PML) along the *x*‐, *y*‐, and *z*‐axes. The mesh grid was defined with a spacing of 2 nm in the *x*‐ and *y*‐directions, and 4 nm in the *z*‐direction. Since the R6G molecular thin film fabricated in the experiment has a thickness of ≈40 nm, the electric field intensity distribution was calculated along the *z*‐axis from 0 to 40 at 4 nm intervals, and then averaged.

The radiative decay rate enhancement factor is obtained by calculating the emitted power enhancement of a dipole source near the AuNR.^[^ ^10^ ^]^ For a simulation of randomly distributed emitters, the radiated power generated by a single dipole along the *x*‐, *y*‐, and *z*‐axes is obtained through three separate simulations, and the results are averaged. The radiative power of the dipole source is measured by recording the amount of radiation leaving the system using a transmission box monitor that surrounds the entire simulation domain. The boundary conditions were set to PMLs along the *x*‐, *y*‐, and *z*‐axes. The mesh grid was defined with a uniform spacing of 2 nm along all three axes.

The scattering spectrum of a single AuNR is calculated by sweeping the incident light wavelength in 1 nm increments, with the light incident at a 5^°^ angle and linearly polarized along the *x*‐ and *y*‐axes. The boundary conditions were set to PMLs along the *x*‐, *y*‐, and *z*‐axes. The mesh grid was defined with a spacing of 5 nm along all three axes. The scattered power is measured using a box monitor surrounding a region with an *x*‐ and *y‐*span of 600 nm and a *z*‐span of 200 nm. The gold nanorod was placed at the center of the box monitor.

### ThunderSTORM Image Reconstruction

For SMLM reconstruction, the ThunderSTORM plug‐in module for ImageJ^[^ ^31^ ^]^ was employed. The raw image sequence consisted of 6000 image frames, each with 1024 × 1024 pixels and a pixel size of 75 nm. The following ThunderSTORM fitting parameters were used for image reconstruction: a wavelet filter and a B‐Spline (order 3, scale 2). For approximate localization of molecules, the local maximum method was used with a standard deviation (Wave.F1) and 8‐neighborhood connectivity. For sub‐pixel localization, the point spread function (PSF) model was set to integrated Gaussian with a fitting radius of 4 pixels. The fitting method was weighted least squares with an initial sigma of 6 pixels. For visualization, a normalized Gaussian rendering method with a magnification factor of 25 was applied. No explicit post‐processing clustering or grouping filters were applied; however, the broad fitting parameters (initial sigma = 6 pixels, fitting radius = 4 pixels) were chosen to minimize overfitting and reduce redundant localizations of slowly blinking fluorophores.

## Conflict of Interest

The authors declare no conflict of interest.

## Supporting information



Supporting Information

## Data Availability

The data that support the findings of this study are available from the corresponding author upon reasonable request.
