# Supplementary material for: Unveiling Exciton‐Plasmon Polariton Coupling Regions via Polarization‐Enhanced Optical Nanoscopy
Source: Adv Sci (Weinh). 2025 Aug 11;12(41):e07822. doi: 10.1002/advs.202507822 (PMC12591100; doi:10.1002/advs.202507822)
Supplement: Supplementary file 1 — Supporting Information [file ADVS-12-e07822-s001.pdf]

Supporting Information

Unveiling Exciton-Plasmon Polariton Coupling Regions via Polarization-Enhanced Optical Nanoscopy

*Bin Chan Joo, Dong Hee Park, Kyu Ri Choi, Yeon Ui Lee**

Contents:

S1. Excitation and emission spectra of R6G

S2. SEM images showing multiple AuNRs

S3. Calculated electric field intensity distribution around an AuNR

S4. Fluorescence intensity time trace

S5. Fourier ring correlation (FRC) analysis

S6. The reproducibility of the localization improvement in P-SMLM

S7. Stepwise fluctuations characteristic of single emitters

S1. Excitation and emission spectra of R6G

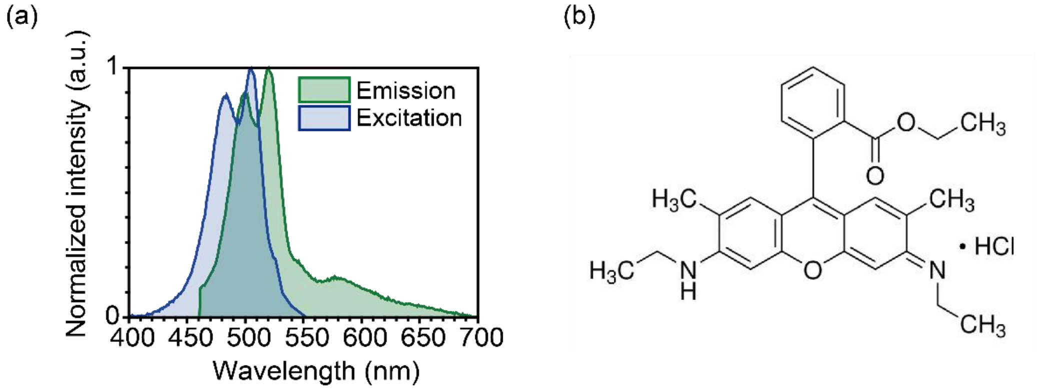

Figure S1 | (a) Excitation and emission spectra of R6G thin film with a thickness of 40 nm. (b) Molecular structure of R6G used in the experiment.

Figure S1a shows the measured excitation and emission spectra of the R6G (**Figure S1b**) thin film. The fluorescence spectra are measured using a fluorometer (Horiba FluoroMax Plus) combined with an optical microscope (Olympus BX53) equipped with a 100× objective lens (NA 0.80). For the emission spectrum, the excitation wavelength is fixed at 420 nm, with emission measured from 450 to 750 nm. For the excitation spectrum, the emission wavelength is fixed at 650 nm, measuring the excitation range from 400 to 510 nm. The fluorescence signal is collected after passing through a 450 nm dichroic mirror. The exposure time is 0.2 s, and the spectra are obtained by averaging five consecutive measurements.

S2. SEM images showing multiple AuNRs

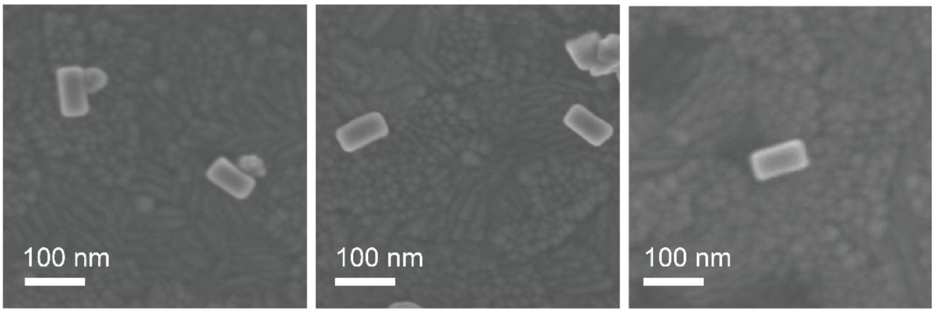

Figure S2 | SEM images of AuNRs used in the experiment. The average dimensions of the gold nanorods are approximately 75 nm × 25 nm.

S3. Calculated electric field intensity distribution around an AuNR

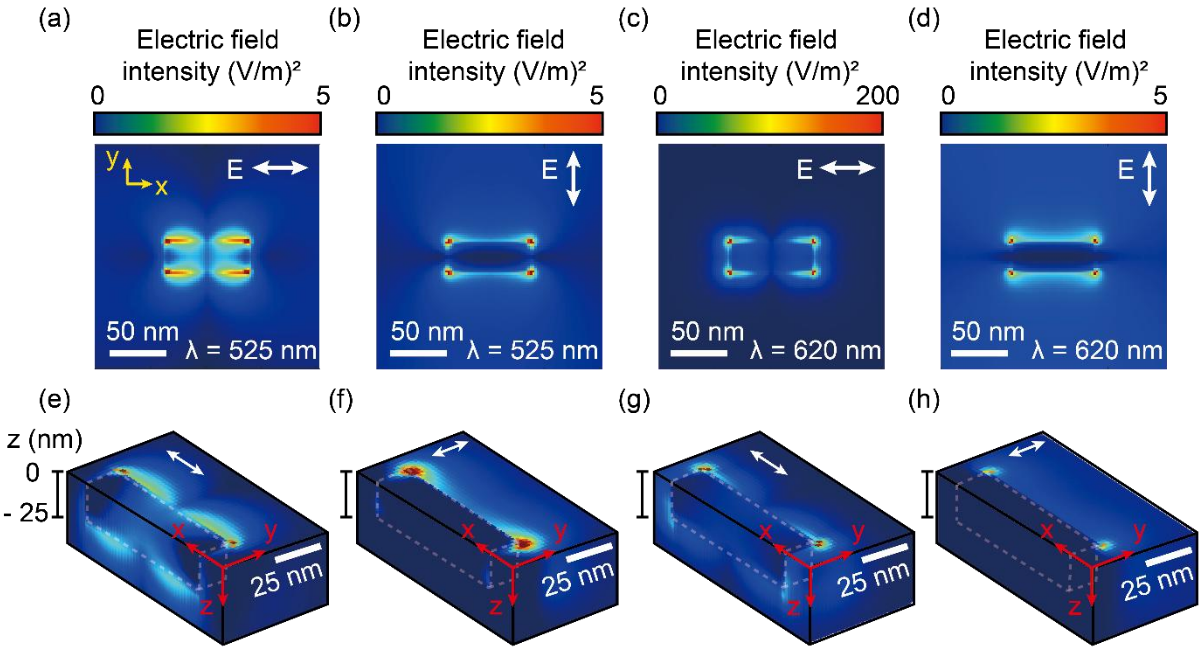

Figure S3 | Calculated electric field intensity distribution around an AuNR, excited by normally incident *x*-polarized and *y*-polarized plane waves with λ = 525 and 620 nm. (a-d) The electric field intensity distribution is calculated by averaging the electric field intensities over *xy*-plane power monitors along the *z*-axis at z = 4 nm intervals. (e-f) Three-dimensional spatial distribution of the calculated electric field intensity near an AuNR. The white arrows show the polarization of the incident plane wave.

To identify the spatial region where the LSPPs, generated by an AuNR, can couple with the excitons of R6G, the near-field intensity distributions are calculated using the three-dimensional FDTD method. The distributions shown in **Figure S3** are obtained by averaging the electric field intensities over *xy*-plane power monitors along the *z*-axis at z = 4 nm intervals at wavelengths of 525 nm and 620 nm. Note that the polarization orientation of the incident beam determines the excitation efficiency of R6G in the exciton-plasmon polariton coupling regime. As shown in **Figure S3**, *x*-polarized light (along the long axis of the AuNR) predominantly excites the longitudinal resonance modes, confining the electric field to ~12 nm at λ = 525 nm and ~6 nm at λ = 620 nm from the AuNR surface. In contrast, *y*-polarized light (along the short axis) excites the transverse resonance mode, with the electric field confined to ~8 nm at λ = 525 nm and ~7 nm at λ = 620 nm. The confined electric fields around a single AuNR increase the local density of state (LDOS) of light, which represents the density of available optical modes in a localized spatial region and directly influences the spontaneous

emission rate of molecular excitons. Enhanced Purcell effect, resulting from the increased LDOS due to LSPPs in the AuNR, amplifies the spontaneous emission decay rate of R6G molecules near the AuNR.

S4. Fluorescence intensity time trace

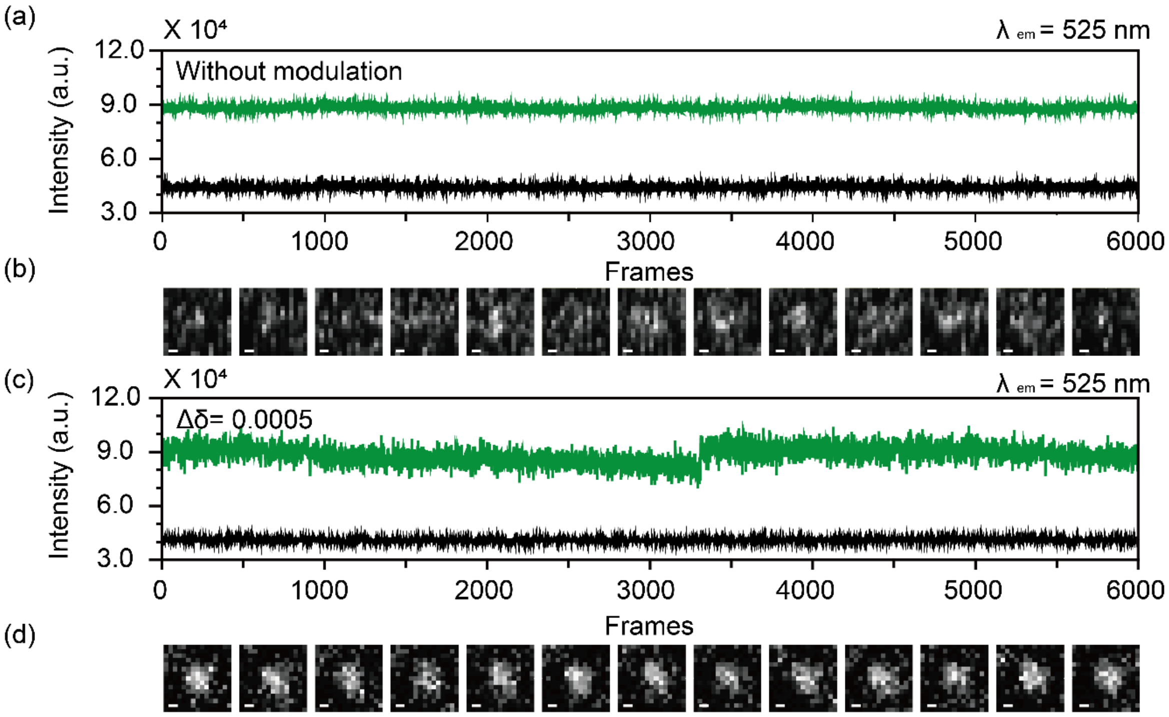

Figure S4| Fluorescence intensity time trace and diffraction-limited fluorescence images at the 500–550 nm emission range for different retardance steps, $\Delta\delta$. (a,b) Fluorescence intensity time trace and diffraction-limited fluorescence images without polarization modulation. (c,d) With polarization modulation, $\Delta\delta = 0.0005$ waves. Green and black solid lines indicate the fluorescence intensity around the AuNR and the background R6G molecules, respectively. (b,d) The fluorescence intensity images represent 12 example frames selected from the image sequence—specifically, the 1st, 500th, 1000th, ..., and 6001st frames out of a total of 6001 frames, with a scale bar: 170 nm.

Figure S4 shows the fluorescence intensity time trace of R6G molecules near a single AuNR at emission wavelengths of λ = 500–550 nm. The PMT exposure time is 200 ms per frame, synchronized with the LCVR modulation signal. Background signals are measured in regions away from any AuNRs. Fluorescence intensity is averaged over a 10 × 10 pixels region. Maximum emission intensity is observed when the AuNR is aligned parallel to the incident beam polarization, indicating strong plasmon polariton interaction with nearby R6G molecules and resulting in enhanced emission intensity. The diffraction-limited fluorescence images in **Figure S4b** and **S4d** represent the pixels used for tracking emission intensity over time for fluorescence intensity measurements. The observed polarization dependence reflects a combination of molecular dipole orientation effects and polarization-induced redistribution of local excitation field near AuNR. Polarization modulation improves contrast and enhances

fluorescence blinking signals. These high-contrast, diffraction-limited images and LCVR-modulated blinking signals enable efficient molecular localization during image reconstruction.

S5. Fourier ring correlation (FRC) analysis

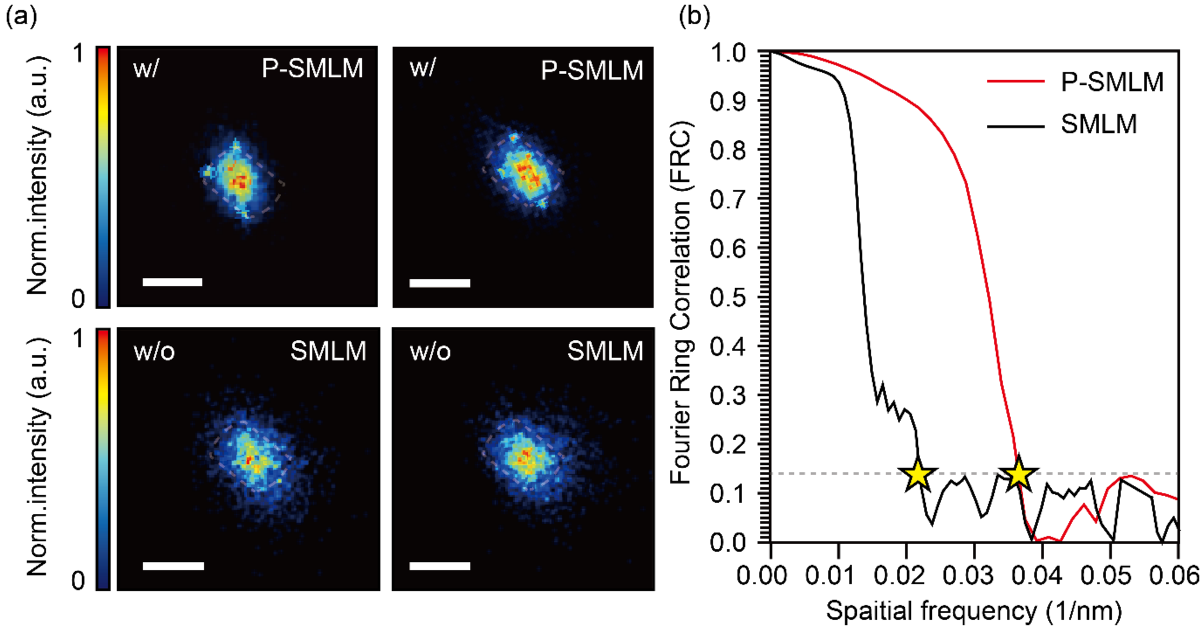

Figure S5 | Fourier ring correlation (FRC) analysis for spatial resolution estimation of the reconstructed SMLM images with and without polarization modulation. (a) For FRC analysis, 3,000 randomly selected subframes were divided into two independent datasets, each used to reconstruct an SMLM image for resolution estimation. (b) The FRC curves were calculated from two independently reconstructed images with (red) and without (blue) polarization modulation. The gray dashed line represents the 1/7 threshold criterion used to estimate the spatial resolution in single-molecule localization microscopy. The intersection of the FRC curve with the threshold indicates a spatial resolution of approximately 26 nm and 41 nm, respectively, with and without polarization modulation. scalebar: 85 nm

S6. The reproducibility of the localization improvement in P-SMLM

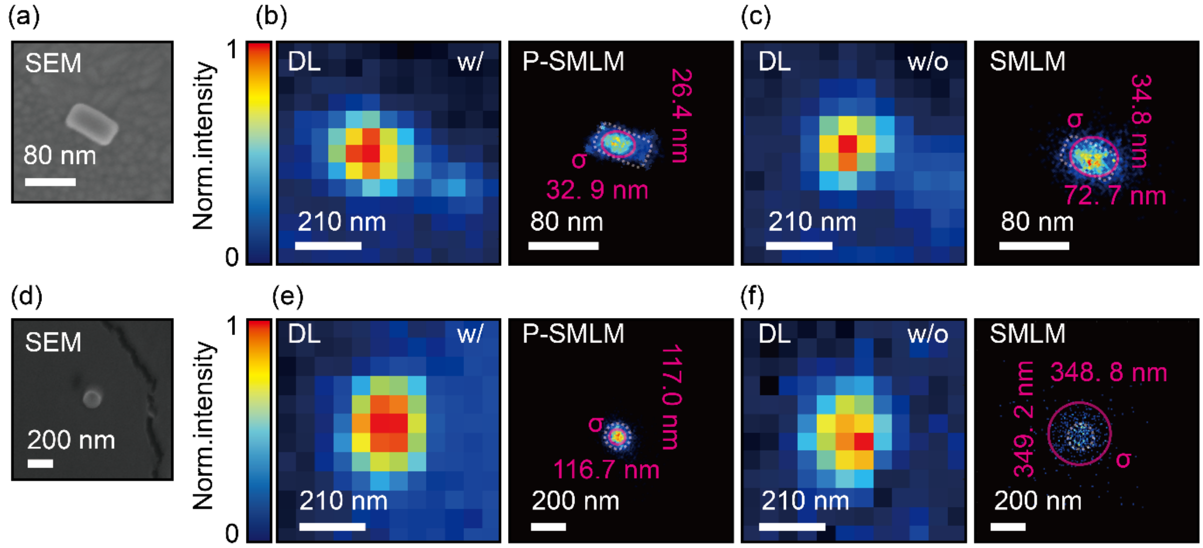

Figure S6 | Visualization of exciton-plasmon polariton interaction regimes near different types of Au nanoparticles. (a) SEM image of the 1:3 aspect ratio AuNR. (b-c) Diffraction-limited (DL), P-SMLM, and conventional SMLM images of exciton-plasmon polariton interaction zones for the AuNR-R6G composite structure at $\lambda = 525$ nm. (d) SEM image of the Au nanocolloid. (e-f) DL, P-SMLM, and conventional SMLM images of exciton-plasmon polariton interaction zones for the Au nanocolloid-R6G composite structure at $\lambda = 525$ nm. The standard deviation (σ) represents the relative hot spot size or the localization accuracy of the centroid position of a hot spot.

S7. Stepwise fluctuations characteristic of single emitters

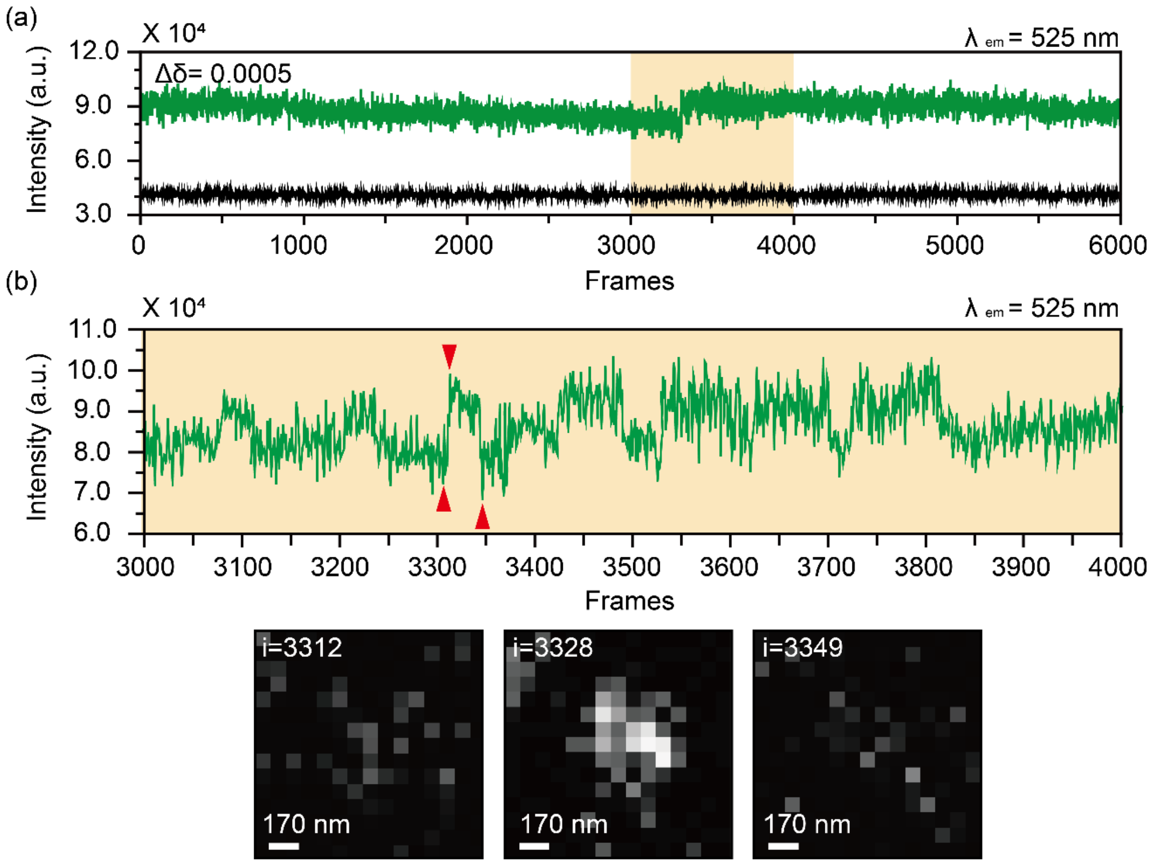

Figure S7 | Fluorescence intensity time traces showing polarization-modulation-induced blinking behavior of individual R6G molecules. Time traces of fluorescence intensity collected from a 3 × 3 pixel region centered near a single AuNR under polarization modulation conditions. The traces show discrete stepwise on/off transitions, indicative of single-molecule blinking behavior. These results support the validity of single-molecule localization analysis despite the high emitter density. The fluorescence time traces exhibit two-level blinking behavior with discrete transitions between ON and OFF states. The absence of intermediate intensity levels and the temporal sparsity of ON events indicate that the signal originates from a single fluorophore, rather than overlapping emitters.

Figure S7 confirms that despite the high density of fluorophores, polarization modulation introduces sufficient spatiotemporal sparsity to enable effective single-molecule localization.
